# Advocacy to End Sexual Harassment: Voices From Women in Cardiology

**DOI:** 10.1016/j.jaccas.2021.04.029

**Published:** 2021-05-19

**Authors:** Kamala P. Tamirisa, Annabelle Santos Volgman, Purvi Parwani, Gina P. Lundberg

**Affiliations:** a Texas Cardiac Arrhythmia Institute, Dallas, Texas, USA; b Department of Internal Medicine/Cardiology, Rush University Medical Center, Chicago, Illinois, USA; c Department of Internal Medicine/Cardiology, Loma Linda Medical Center, Loma Linda, California, USA; d Department of Internal Medicine/Cardiology, Emory University School of Medicine, Atlanta, Georgia, USA

**Keywords:** cardiology, gender harassment, sexual discrimination

*Sexual harassment is a violation of safety culture.*

—The Joint Commission

Sexual harassment is a global issue affecting an individual’s work performance by creating an intimidating and unsafe environment. In this article, we address the issues specific to cardiology, provide a brief background on the current regulations in academic medicine, and review some of the programs being championed by the American College of Cardiology (ACC) leadership.

## Sexual Harassment Definition

Sexual harassment is not universally defined and sometimes difficult to separate from the spectrum of other misconduct, including gender harassment, gender bias, bullying, and unprofessional behavior. The 2018 Statement from the National Academies of Science, Engineering, and Medicine Report on Sexual Harassment of Women defined sexual harassment as inclusive of verbal and nonverbal cues, exclusion, sexual assault, and coercion (1). Harassing behavior can be either direct (targeted at an individual) or ambient (a general level of sexual harassment in an environment). Several factors contribute to sexual harassment, including perceived acceptance for misconduct, hierarchical structures with power differentials, superficial compliance with Title VII or Title IX, a permissive environment, and lack of effective and diverse leadership.

## Cardiology-Specific Data

Only 13% to 14% of all practicing cardiologists are women (2). Medical residents have perceived the specialty of cardiology as being unwelcoming to female individuals, unsupportive of families, and a “toxic climate” for trainees, including sex-based discrimination (3). Based on the ACC third decennial Professional Life Survey, two-thirds of women in cardiology reported experiencing sex- and parenting-related discrimination (4). According to a cross-sectional German study using an online questionnaire, 32% of female cardiologists report sexual harassment in the workplace (5). Nearly 62% of female cardiologists in the United Kingdom reported discrimination, mostly related to gender and parenting responsibilities. In the same study, one-third of women reported experiencing sexual harassment (6). Women are more likely to report sexual harassment by colleagues (12% women vs. 1.3% men) based on the online survey conducted by the ACC in an international survey (7). An online survey was conducted under the direction of the ACC Women in Cardiology Leadership Council to assess perspectives of fellows in training regarding professional and personal elements that influenced cardiology subspecialty career choices. Although this survey was not specifically focused on the issue of sexual harassment alone, women were more likely to perceive sexual harassment as one of the negative attributes of choosing a career path in interventional cardiology (8).

## Current Legislation

The details of the current protection acts are beyond the scope of the present paper. However, it is important to know the current context and its weaknesses. Title IX of the Education Amendments of 1972 prohibits any sex discrimination, including sexual harassment, in any education program or activity receiving federal financial assistance (9). Title VII of the 1964 Civil Rights Act is based on illegal sex discrimination and the target’s employment status (10). Although all academic institutions are outwardly compliant with the legal requirements of Title IX, some academic institutions have not completely enforced extensive compliance with the policies (11). Reporting is a weak link in addressing sexual harassment, based on the assumptions that victims can report without retaliation and that reporting will lead to serious investigation with corrective action (1). For example, retaliation for reporting was openly witnessed in a settled legal case against a practicing cardiologist (12). There may be many reasons for the lack of meaningful compliance with the Title IX act, such as institutional reputation (1). Examples of organizational tolerance of sexual misconduct include a sexual harassment case against a renowned cardiologist and endowed chair at Yale University and another case against the University of Southern California by an Internal Medicine resident who is currently a cardiology fellow (13,14). Lack of action and dismissal of complaints of sexual harassment as a rare phenomenon could increase stigmatization, lead to underreporting, and perpetuate a permissive environment (15,16).

In cardiology, only 16.1% of affected women sought help for sexual harassment, according to the online ACC survey (6). Indirect reporting channels outside the department, such as an ombudsperson (rather than direct reporting to the program director/hierarchical ladder if the victim so chooses), could be a solution to this concern (17). Investing in diversity in academic institutions and employee stakeholders with a horizontal restructuring of the leadership, a policy of zero tolerance, and bystander training could help mitigate sexual harassment (1). It is important to track data to evaluate the efficacy of any implemented programs. One such example is the Royal Australasian College of Surgeons, which undertook a multi-year initiative with research and an action plan to end a perceived climate of discrimination and sexual harassment (18).

## ACC Efforts

ACC took active leadership in addressing sexual harassment. The Ad Hoc Committee on Women in Cardiology conducted a Professional Life Survey to assess the career decisions of women and men in cardiology. Although it was not specifically geared to address sexual harassment, the survey showed that most women (71%) reported gender discrimination (19). The ACC Diversity and Inclusion Task Force, founded in 2017, made recommendations, including intensified efforts to identify and overcome gender biases toward zero-tolerance policy (20). The ACC leadership in 2018 laid the foundation to address sexual harassment in cardiology with a directive of moving the needle from #MeToo to #MeNeither, with recommendations for sexual harassment training and calling for the implementation of a code of conduct (21). As part of the ACC’s Leadership Forum in 2018, a special session titled “Me Too, But Now What?” was conducted. The session’s goal was to educate incoming ACC leaders about ways to enforce a safe and accountable workplace for all. The ACC’s Women in Cardiology Section hosted a dedicated session on harassment in cardiology at the 2018 ACC meeting. The attendee conduct policy for all ACC events was made standard. The ACC Code of Ethics clearly states, “A member shall not engage in any form of discrimination, harassment, or retaliation, including but not limited to, sexual harassment; harassment on the basis of any other protected characteristic; denial of opportunity or unfair treatment resulting from bias or prejudice; or action taken with the intent to negatively impact another individual based on the reporting of an act of discrimination or harassment, where the likely result of such action would be an objective material detriment to the individual who reported such an act of discrimination or harassment and/or his or her career” (22).

As the ACC and American Heart Association try to attract more diversity in cardiology and improve gender equity, we must continue to address the hurdles that limit progress and eliminate misconduct, discriminatory behaviors, and sexual harassment. With that intention, the American Heart Association and the ACC Foundation Consensus Conference on Professionalism and Ethics report recommended that the educators have an essential role in eliminating sexual harassment and gender bias in medical schools and training programs. They emphasize accountability among the educators and the adoption of prevention strategies to ensure confidential reporting and transparent investigations.

As dedicated educators, clinicians, and researchers, we need to continue to reform to eradicate sexual harassment and discrimination and rebuild a more just and equitable cardiology culture.

## Funding Support and Author Disclosures

Dr. Volgman has received grants from MSD/Bayer Virtual Global Advisory Board Member, Bristol Myers Squibb Foundation Diverse Clinical Investigator Career Development Program (DCICDP), Novo Nordisk Virtual Advisory Board on Healthcare Disparities, Novartis Clinical Trial, NIH Clinical Trials, Apple Inc. stock. Dr. Parwani has served as a Guest Editor for *JACC: Case Reports*. Dr. Lundberg has served as social media consultant for *JACC: Case Reports*. All other authors have reported that they have no relationships relevant to the contents of this paper to disclose.
